# Combination of Transcriptomics and Metabolomics Analyses Provides Insights into the Mechanisms of Growth Differences in Spotted Seabass (*Lateolabrax maculatus*) Fed a Low-Phosphorus Diet

**DOI:** 10.3390/metabo14080406

**Published:** 2024-07-25

**Authors:** Nan Jin, Ling Wang, Kai Song, Kangle Lu, Xueshan Li, Chunxiao Zhang

**Affiliations:** 1 State Key Laboratory of Mariculture Breeding, Fisheries College, Jimei University, Xiamen 361021, China; 202211908041@jmu.edu.cn (N.J.); lingwang@jmu.edu.cn (L.W.); songkai@jmu.edu.cn (K.S.); lukangle@jmu.edu.cn (K.L.); 2 Xiamen Key Laboratory for Feed Quality Testing and Safety Evaluation, Fisheries College, Jimei University, Xiamen 361021, China

**Keywords:** *Lateolabrax maculatus*, RNA-seq, metabolomics, growth

## Abstract

To analyze the potential mechanisms of growth differences in spotted seabass (*Lateolabrax maculatus*) fed a low-phosphorus diet, a total of 150 spotted seabass with an initial body weight of 4.49 ± 0.01 g were used (50 fish per tank) and fed a low-phosphorus diet for eight weeks. At the end of the experiment, five of the heaviest and five of the lightest fish were selected from each tank as fast-growing spotted seabass (FG) and slow-growing spotted seabass (SG), respectively, and their livers were analyzed by metabolomics and transcriptomics. The hepatic antioxidant capacity of the FG fed a low-phosphorus diet was significantly higher than that of the SG. A total of 431 differentially expressed genes (DEGs) were determined in the two groups, and most of the DEGs were involved in metabolism-related pathways such as steroid biosynthesis, glycolysis/gluconeogenesis, and protein digestion and absorption. Substance transport-related regulators and transporters were predominantly up-regulated. Furthermore, a large number of metabolites in the liver of FG were significantly up-regulated, especially amino acids, decanoyl-L-carnitine and dehydroepiandrosterone. The integration analysis of differential metabolites and genes further revealed that the interaction between protein digestion and absorption, as well as phenylalanine metabolism pathways were significantly increased in the liver of FG compared to those of the SG. In general, FG fed a low-phosphorus diet induced an enhancement in hepatic immune response, substance transport, and amino acid metabolism. This study provides new information on genetic mechanisms and regulatory pathways underlying differential growth rate and provides a basis for the foundation of efficient utilization of low-phosphorus diets and selective breeding programs for spotted seabass.

## 1. Introduction

Spotted seabass (*Lateolabrax maculatus*) is an economically important fish that is widely cultured in China, with an annual production of 21.97 million tons in 2022 [1]. In order to ensure the further development of the spotted seabass aquaculture industry, urgent action is required to improve the nutrient requirements database of this species. Phosphorus is one of the key nutrients essential for animal growth. The discharge of unused phosphorus into the water during the aquaculture process will exacerbate the water pollution problem. An effective method for alleviating eutrophication in the water is to decrease the phosphorus content in the feed. Meanwhile, the consumption of low-phosphorus feed by spotted seabass causes a significant decrease in their growth rate compared to fish fed diets with appropriate phosphorus levels [2]. Therefore, it is of great significance to study the mechanisms by which fish can mitigate or adapt to the adverse effects of low-phosphorus feed [3].

In the process of aquaculture, the issue of growth disparities frequently arises due to inadequate fish nutrition [4]. Research has found significant differences in the growth rate of spotted seabass individuals fed a low-phosphorus diet [2]. In addition, some fish demonstrate rapid growth ability under low-phosphorus feed conditions and can even reach the level achieved with normal phosphorus feed [5]. This disparity is not only reflected in growth rate but also encompasses alterations at multiple physiological and metabolic levels. As the primary metabolic organ in fish, the liver plays a pivotal role in growth regulation. The liver is not only involved in synthesizing and metabolizing essential biocompounds such as carbohydrates, lipids, and proteins but also holds a central function in immune responses [6,7]. The efficiency of nutrient uptake and utilization in the liver has a significant effect on the growth and development of fish. Revealing the molecular mechanisms and growth regulatory pathways within the liver is crucial for optimizing fish performance on a low-phosphorus diet.

For elucidating the molecular mechanisms of fish growth, transcriptomics and metabolomics techniques have provided powerful tools [8,9,10,11]. Transcriptomics has been widely used to decipher differential expressed genes (DEGs) related to different biological functions, such as growth [12,13], development [14], and immunity [15,16]. Transcriptomics and metabolomics techniques provide valuable insights into the molecular metabolic mechanisms related to the fish growth rate. For instance, the key regulatory factor of lipid metabolism, *FABP*, is significantly up-regulated in the liver of fast-growing Chinese catfish (*Leiocasis longirostris*) compared to slow-growing individuals [17]. Similarly, significant differences in gene expression related to amino acid metabolism have been observed in the liver of spotted forktail fish (*Ictalurus punctatus*) exhibiting varying growth rates [14]. Furthermore, omics techniques are used to identify 106 DEGs in the muscle of largemouth bass (*Micropterus salmoides*), enabling the screening of candidate genes and metabolites related to growth. In addition, the comprehensive analyses of transcriptomics and metabolomics have become an effective analytical method to explore the relationship between relevant genes and metabolites [18,19,20]. Previous studies have indicated that spotted seabass increase protein catabolism [21] and affect the differentiation of osteoblast cells [21] due to their inability to adapt to a low-phosphorus diet. However, the mechanism of how spotted seabass adapt to low-phosphorus environments and exhibit individual growth-rate differences after being fed a low-phosphorus diet is still unclear.

This study aims to explore the liver differences and adaptation mechanisms of spotted seabass under low-phosphorus feeding conditions with different growth rates through transcriptomics and metabolomics analyses. The results provide a theoretical basis for the development of a low-phosphorus diet in fish farming.

## 2. Materials and Methods

### 2.1. Feed and Feeding Experiment

The low-phosphorus feed (Table 1) was produced following the protocol described by Guo et al. [2].

The feeding experiment was conducted in the recirculating aquaculture system at Jimei University in Fujian Province, China. The spotted seabass used in this experiment is from a fish hatchery in Zhangzhou. The fish were fed a commercial feed for two weeks before the start of the formal experiment. A total of 150 healthy fish with a mean body weight of 4.49 ± 0.01 g were distributed randomly into three identical fiberglass tanks (200 L each, with 50 fish per tank) at the end of the acclimation period. The fish were fed ad libitum twice a day (8:00 and 17:00) for 8 weeks. During the feeding test, the water conditions were kept as follows: dissolved oxygen > 6.5 mg/L; water temperature between 26 and 27 °C; and pH ranging from 6.9 to 7.2.

### 2.2. Sample Collection

After the end of feeding, 150 fish were fasted for 24 h and then weighed and sampled. The selected fish were anesthetized with 0.1 g/L MS-222 (Sigma, Ronkonkoma, NY, USA). The weight of the collected liver and body length were recorded for growth performance statistics. The top 10% and bottom 10% of the body weight (BW) data were selected as two groups for fast-growing spotted seabass (FG) and slow-growing spotted seabass (SG). Ten liver samples were randomly selected from each group for liver metabolomics and transcriptomics analyses. The liver samples of five fish were collected and placed in 2.0 mL EP tubes, immediately frozen in liquid nitrogen, and then stored in a −80 °C freezer for subsequent liver biochemical analysis and RT-qPCR analysis.

### 2.3. Liver Biochemistry Analysis

Liver samples were carefully weighed, homogenized in 9 ice-cold volumes (*w*/*v*) of 0.86% NaCl, and then centrifuged at 2000 rpm for 10 min for antioxidant capacity analysis. The protein concentration was measured using a BCA protein assay kit (Solarbio, Beijing, China). The capacity of T-AOC (A015-2-1) and the activities of SOD (A001-3), GSH-PX (A005-1), and CAT (A007-1-1) in the liver were measured using commercial assay kits (Jiancheng Biotech. Co., Nanjing, China) according to the manufacturer’s instructions.

### 2.4. Quantitative Real-Time PCR

Total RNA from the liver was extracted and subjected to RT-qPCR following the procedures described in our previous study [22]. The gene primers used for RT-qPCR are detailed in Table 2. The relative expression levels of genes were determined using the 2^−ΔΔCt^ method [23].

### 2.5. Transcriptomics Sequencing (RNA-Seq) Analysis

Liver samples were utilized for total RNA extraction, followed by genomic DNA removal, RNA purification, and quality assessment. RNA-seq libraries were then generated using a commercial kit and sequenced on an Illumina platform. After quality control, the clean reads were annotated against the *Lateolabrax maculatus* genome. The DEGs in the FG and SG were identified and analyzed for GO term enrichment and pathway functions. Furthermore, the transcriptional profiles of immune and substance transport-related DEGs were investigated. The transcriptomics quality was validated through qPCR analysis of DEGs markers (Table 2).

### 2.6. Metabolomics Analysis

Following pretreatment of the liver samples, the extracted metabolites were analyzed using liquid chromatography-tandem mass spectrometry (LC-MS/MS). Subsequently, the metabolomics data underwent quality control and were annotated with the appropriate database. Then, the metabolomics data were standardized through autoscaling before undergoing multivariate statistical analysis using orthogonal partial least squares discriminant analysis (OPLS-DA), with a significance level set at *p* < 0.05 and VIP > 1.0. A non-parametric test, specifically the Mann–Whitney–Wilcoxon test, was utilized for comparing the two groups. Differential metabolites for the FG and SG were identified separately, followed by an analysis of the pathways of these differential metabolites and further identification of metabolite biomarkers.

### 2.7. Comprehensive Transcriptomics and Metabolomics Analyses

Briefly, the liver distribution algorithm facilitated the identification of co-enriched pathways via a Kyoto Encyclopedia of Genes and Genomes (KEGG) enrichment analysis of both differential gene sets and metabolic sets. Furthermore, by calculating the correlation between differential metabolites and genes, the interactions among molecules were explored, ultimately resulting in the construction of interaction diagrams for various pathways based on their upstream and downstream relationships.

### 2.8. Statistical Analysis

All statistical analyses were performed on IBM SPSS 22.0 with an independent sample *t*-test, and the results were marked as: * means *p* < 0.05; ** means *p* < 0.01. Quantitative data were reported using the mean ± standard error.

## 3. Results

### 3.1. Differences in Growth Performance

**Figure 1:**
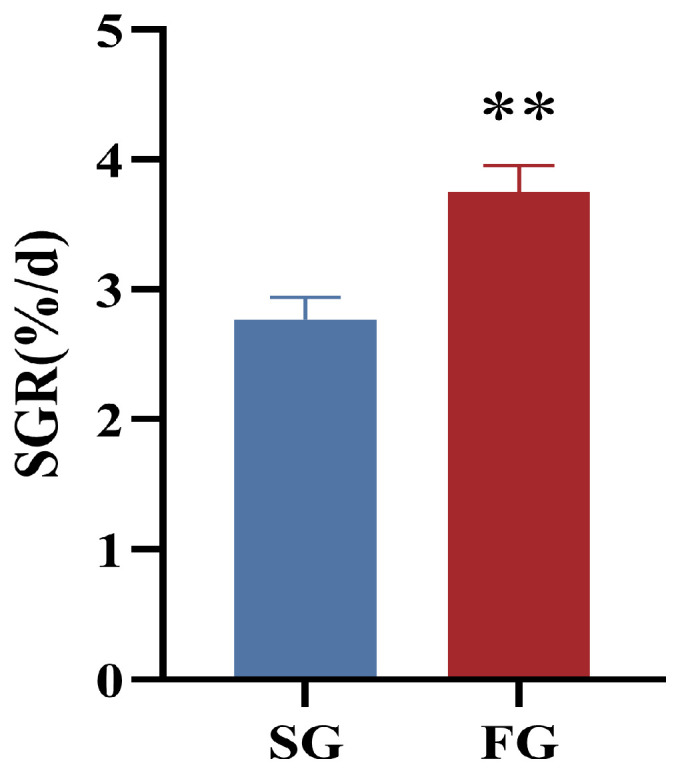
The specific growth rate (SGR, %/d) of fast-growing spotted seabass (FG) and slow-growing spotted seabass (SG). “**” indicates highly significant differences between FG and SG (*t*-test, *p* < 0.01). Specific growth rate (%/d) = (ln final body weight − ln initial body weight)/time × 100.

The specific growth rate of FG (3.75 ± 0.20%/d) was extremely significantly higher than that of SG (2.77 ± 0.17%/d, *p* < 0.01; Figure 1).

### 3.2. Oxidative Stress-Related Parameters in the Liver

**Figure 2:**
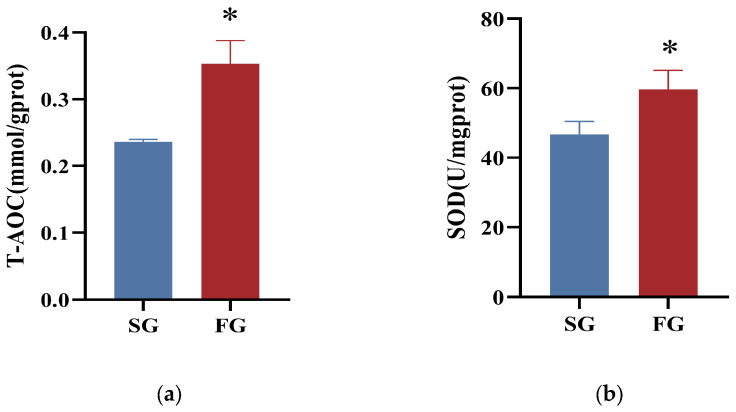

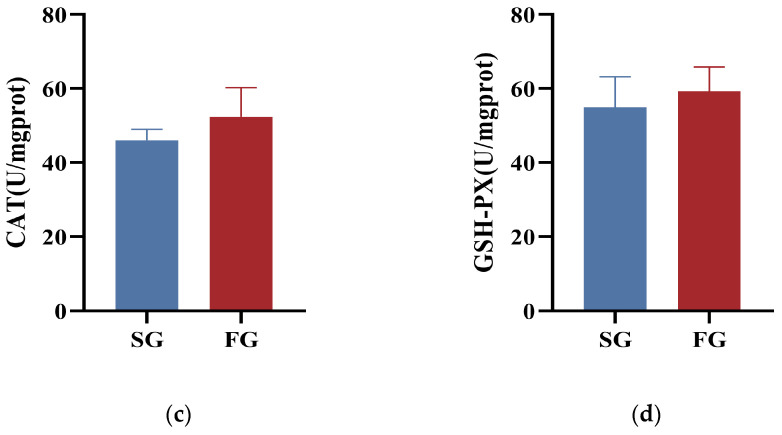
The liver antioxidant capacity of fast-growing spotted seabass (FG) and slow-growing spotted seabass (SG). (**a**) T-AOC: total antioxidant capacity; (**b**) CAT: catalase; (**c**) SOD: superoxide dismutase; (**d**) GSH-PX: glutathione peroxidase. “*” indicates significant differences between FG and SG (*t*-test, *p* < 0.05).

The hepatic SOD activity and T-AOC content of SG were significantly lower than those of FG (*p* < 0.05). In addition, there was no significant difference in activities of CAT or GSH-PX between the two groups (*p* > 0.05; Figure 2).

### 3.3. Expression of Genes Associated with Inflammatory Response

**Figure 3:**
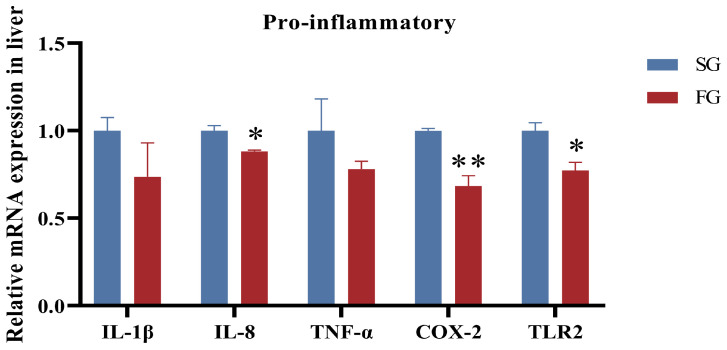
The expression levels of genes involved in innate immunity in the liver of fast-growing spotted seabass (FG) and slow-growing spotted seabass (SG). *IL-1β*: interleukin-1β; *IL-8*: interleukin-8; *COX-2*: cyclooxygenase-2; *TNF-α*: tumor necrosis factor α; *TLR2*: toll-like receptor 2. “*” indicates significant differences between FG and SG (*t*-test, *p* < 0.05); “**” indicates highly significant differences between FG and SG (*t*-test, *p* < 0.01).

Gene expression associated with inflammatory response was assessed in the liver of fish with different growth rates. Compared with SG, the mRNA expression of *IL-8*, *COX-2*, and *TLR2* was significantly lower in FG (*p* < 0.05), whereas the mRNA expression of *IL-1β* or *TNF-α* was not significant (*p* > 0.05; Figure 3).

### 3.4. Transcriptomics Analysis

#### 3.4.1. Transcriptomics Sequence Evaluation and Annotation

The analysis of transcriptomics quality revealed that the percentage of Q30 bases ranged from 95.41% to 95.65% for both FG and SG (Table 3). Clean reads from each sample were aligned with the reference genome, achieving alignment efficiencies ranging from 97.57% to 97.87%. This indicated that high-quality data were used for subsequent analysis (Table 4).

#### 3.4.2. Significant Functional Enrichment Analysis of DEGs

**Figure 4:**
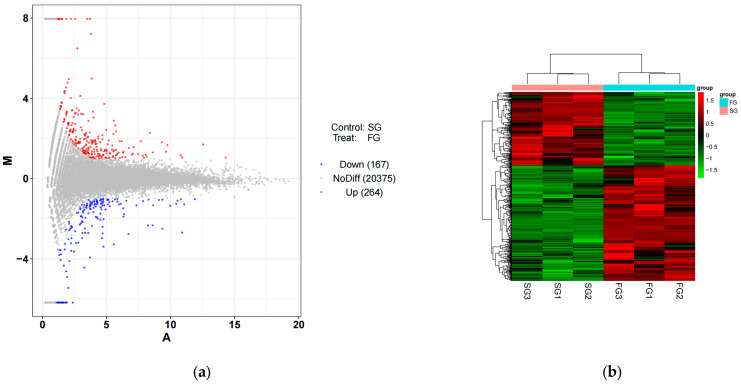

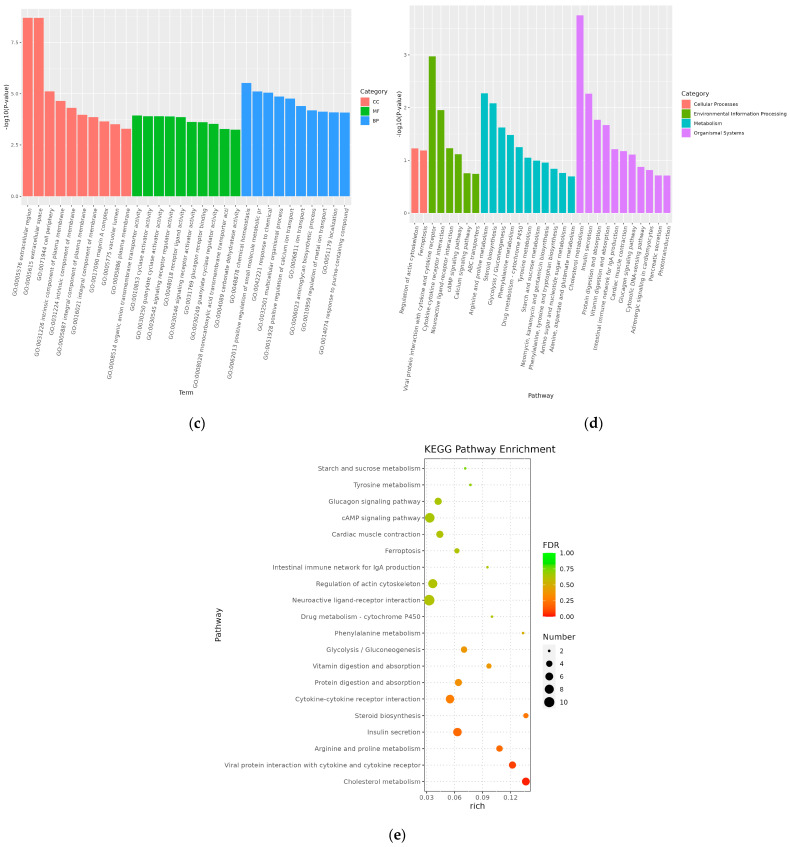
Transcriptome comparison in the liver of fast-growing spotted seabass (FG) and slow-growing spotted seabass (SG). (**a**) Volcano plot of DEGs. (**b**) Cluster heatmaps of DEGs. (**c**) Gene ontology (GO) enrichment of DEGs. (**d**) Classification map of DEGs enrichment pathways. (**e**) The KEGG enrichment scatter plot of DEGs.

A total of 431 growth-related DEGs were found in the two comparison groups (Figure 4a). Among them, compared with SG, 264 DEGs were up-regulated and 167 DEGs were down-regulated in the liver of the FG. The DEGs mainly participated in cytoskeleton generation, immunity, and signal transduction, including *CCR3*, *GCK*, *GAPDH*, and *SLC7A7* (Table 5). The further hierarchical cluster analysis of the DEGs revealed that the expression patterns differed significantly among the pairwise comparison groups, while those DEGs remained similar within different varieties within each group, indicating the reliability of our experimental data (Figure 4b).

The GO and KEGG enrichment analyses were conducted to obtain the potential functions and metabolic pathways of DEGs. The GO analysis classified 431 DEGs into biological process (BP), molecular function (MF), and cellular component (CC) categories. Among these, 79.5% of DEGs were enriched in BP, 7.8% of DEGs in CC, and 12.5% of DEGs in MF. Biological processes were enriched in terms of biological process, cellular process, biological regulation, and multicellular organismal process. Cellular components were enriched in terms of cellular component, cellular anatomical entity, and cytoplasm. The molecular function and binding were enriched in terms of their respective categories. DEGs exhibited significant enrichment in specific categories (Figure 4c). DEGs were significantly enriched in chemical equilibrium, positive regulation of small molecule metabolism, positive regulation of chemical reactions, and positive regulation of calcium ion transport in BP. The significantly enriched GO terms within the molecular function category included organic anion transmembrane transport activity, cyclase activator activity, guanylate cyclase activator activity, and signal receptor modulator activity. Under the cellular component category, more genes were assigned to the GO terms of extracellular regions, extracellular spaces, cell peripheries, and intrinsic components of plasma membranes.

The biological pathways of DEGs were identified using KEGG pathway analysis. The DEGs were primarily enriched in four major categories: signal transduction, endocrine system, metabolism, and organismal systems. Compared to the SG group, the significantly enriched pathways of up-regulated DEGs in the FG group were insulin secretion, cytokine receptor interactions, phenylpropanoid metabolism, glycolysis and gluconeogenesis, steroid biosynthesis, as well as protein digestion and absorption (Figure 4d,e).

#### 3.4.3. RT-qPCR Verification

**Figure 5:**
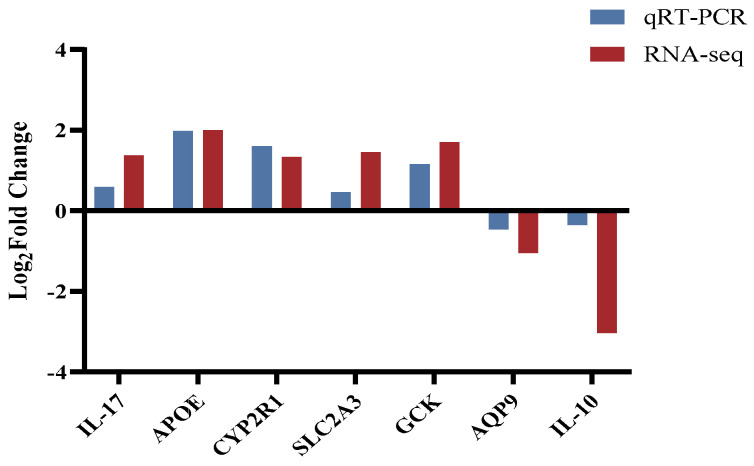
RT-qPCR of DEGs in the liver of fast-growing spotted seabass (FG) and slow-growing spotted seabass (SG). Abbreviations: *IL-17*: interleukin-17; *APOE*: apolipoprotein Eb; *CYP2R1*: vitamin D 25-hydroxylase; *SLC2A3*: glucose transporter 3; *GCK*: glucokinase; *AQP9*: aquaporin 9; *IL-10*: interleukin-10.

Based on an extensive literature review and the results of DEGs identified in this experiment, seven genes related to growth metabolism were randomly selected for real-time fluorescence quantitative PCR to confirm the reliability and accuracy of the transcriptomics data. The genes were interleukin-17 (*IL-17*), apolipoprotein Eb (*APOE*), vitamin D 25-hydroxylase (*CYP2R1*), glucose transporter 3 (*SLC2A3*), glucokinase (*GCK*), aquaporin 9 (*AQP9*), and interleukin-10 (*IL-10*). These genes were involved in neuroactive ligand–receptor interactions, the immune system, carbohydrate metabolism, and the digestive system. Based on real-time fluorescence quantitative PCR data (Figure 5), various indicators were analyzed and calculated using *β-actin* as a reference gene, resulting in a graph. The transcriptomics sequencing data and PCR results of the two groups were consistent, indicating the reliability of the transcriptomics results and suggesting a certain correlation between the screened DEGs and growth.

### 3.5. Metabolomics Analysis

#### 3.5.1. Differential Metabolites (DMs) Analysis

**Figure 6:**
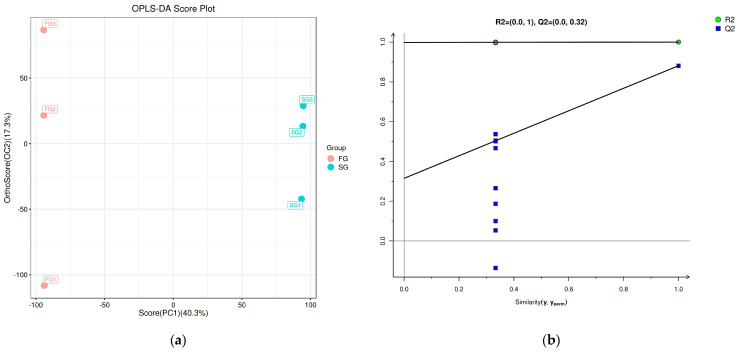

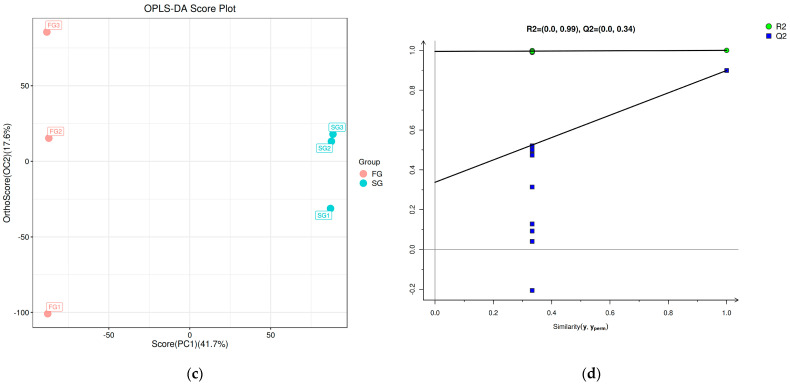
OPLS-DA score chart (**a**,**c**) and OPLS-DA permutation test chart (**b**,**d**) of metabolome profiles in the liver of fast-growing spotted seabass (FG) and slow-growing spotted seabass (SG).

The effect of a low-phosphorus diet on the liver metabolism of spotted seabass with different growth rates was explored through metabolomics analysis. The OPLS-DA score plot and permutation test revealed a significant difference between FG and SG in both positive and negative ionization modes (Figure 6).

#### 3.5.2. Identification and Functional Analysis of Differential Metabolites

**Figure 7:**
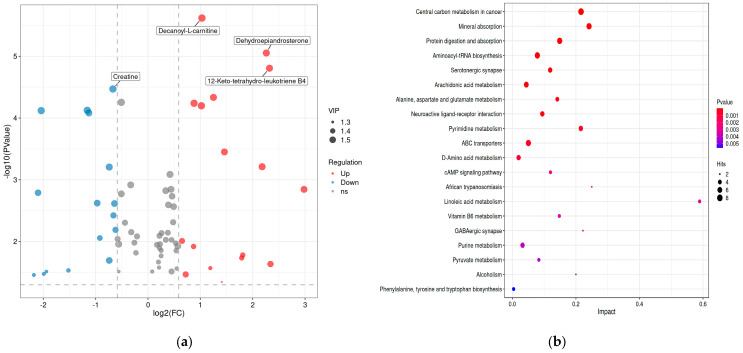
Metabolome comparison in the liver of fast-growing spotted seabass (FG) and slow-growing spotted seabass (SG). (**a**) Volcano map of differential metabolites (DMs). (**b**) KEGG enrichment scatter plot of DMs.

Changes in the metabolic response in the liver of FG and SG were analyzed (Figure 7a). Analysis of multiple variables showed significant variations in metabolic profiles between FG and SG. A total of 70 DMs were identified, with 42 up-regulated DMs and 28 down-regulated DMs found in FG. A clustering heatmap was used to display the overall distribution of metabolites with different abundances in a more intuitive manner. Compared with SG, FG showed significant upregulation of decanoyl-L-carnitine and dehydroepiandrosterone (DHEA) in the liver; creatine was significantly down-regulated (Table 6, Figure 7a).

Further analysis was conducted on the pathways of differential metabolites (Figure 7b). A total of 142 KEGG annotations were obtained from the identified 70 DMs. To further reveal the overall pathway enrichment characteristics of all differential metabolites, an enrichment analysis on the KEGG annotation results was conducted. Among them, the pathways of “Central carbon metabolism”, “Mineral absorption”, “Biosynthesis of plant secondary metabolites” and “Protein digestion and absorption” were dominant in both groups (Figure 7b).

### 3.6. Comprehensive Transcriptomics and Metabolomics Analyses

**Figure 8:**
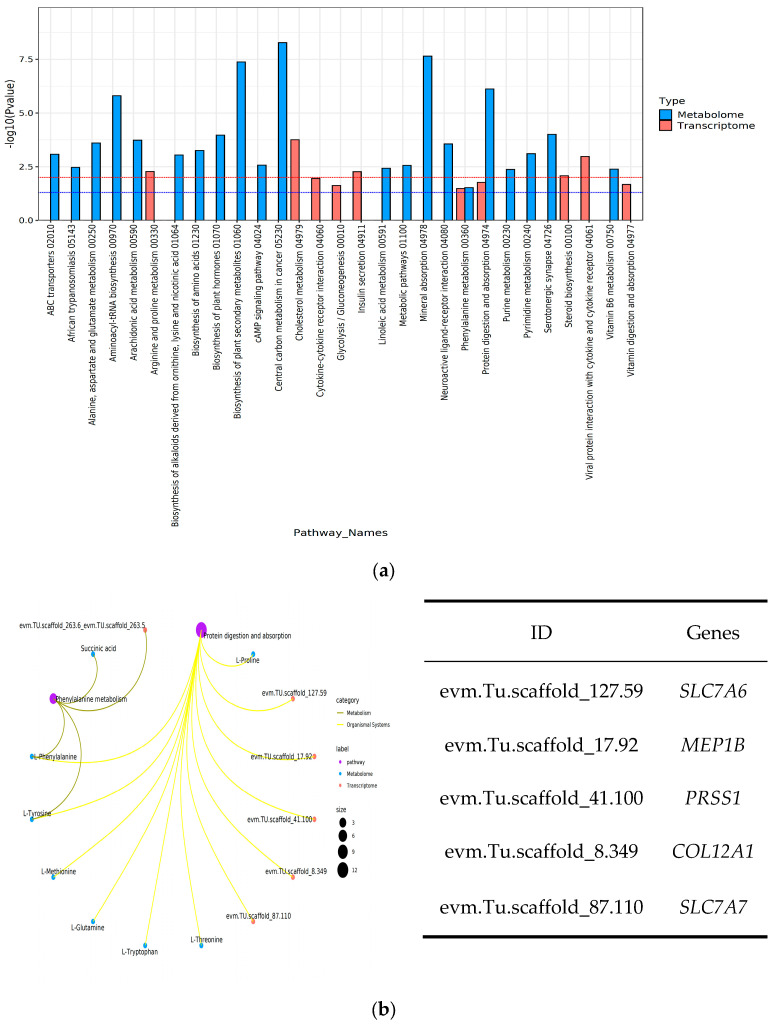
The pathway annotation of differential metabolites in the liver of fast-growing spotted seabass (FG) and slow-growing spotted seabass (SG). (**a**) The number of differential metabolites and differential mRNAs on the enriched pathway. (**b**) The pathway networks between enriched pathways and differential metabolites, as well as differential mRNAs.

According to the integration of transcriptomics and metabolomics, two pathways were significantly enriched in both groups (Figure 8a), including “protein digestion and absorption” and “phenylalanine metabolism”. In the protein digestion and absorption pathway, the expression of *PRSS1*, *MEP1B*, *COL12A1*, and *SLC7A7* genes was significantly up-regulated, while *SLC7A6* gene expression was significantly down-regulated (Figure 8b). The products involved in the regulation included L-Glutamine, L-Methionine, L-Tryptophan, L-Phenylalanine, L-Tyrosine, L-Proline, and L-Threonine. In the phenylpropane metabolic pathway, *AL3B1* gene expression was significantly up-regulated, whereas *OXLA* gene expression was significantly down-regulated. The products involved in regulation included Succinic acid, L-Phenylalanine, and L-Tyrosine.

## 4. Discussion

Growth is one of the most important traits in various aquaculture varieties, which is directly influenced by host genetics, feed, and the environment. Under the same conditions of feeding a low-phosphorus diet, FG exhibited a higher specific growth rate compared to those of SG, which probably indicated some degree of adaptation to low-phosphorus conditions in FG [2]. Thus, elucidating the underlying mechanisms is especially important from both genetic and economic perspectives [24,25]. In this study, the antioxidant capacity in the liver of FG was higher than that of SG through biochemical analysis. Furthermore, transcriptomics and metabolomics analyses have revealed that the liver of FG fed a low-phosphorus diet predominantly modulates nutrient absorption and utilization, immune responses, and energy metabolism pathways, thereby contributing significantly to its enhanced growth. This study is the first report about the molecular mechanism of fast-growing spotted seabass fed a low-phosphorus diet by integrating transcriptomics and metabolomics analyses.

### 4.1. Fast-Growing Spotted Seabass Fed a Low-Phosphorus Diet Displayed Higher Antioxidant Capacity

Antioxidant capacity is closely related to the health status of aquatic animals [26]. SOD is the main antioxidant enzyme that protects animal cells and tissues from oxidative stress [27]. The T-AOC is a comprehensive indicator that reflects the total amount of all antioxidant substances and enzymes in the organism. The higher the value is, the stronger the resistance the organism has to oxidative stress. The results showed that T-AOC and the SOD activity in the liver of SG were significantly lower than those in the FG. This indicated that the liver of SG had a weaker antioxidant capacity. It could be attributed to the close correlation between growth rate and metabolic rate [28]. Furthermore, FG had a high metabolic rate and required more energy and nutrients to support their growth. However, this high metabolic rate generated a large amount of reactive oxygen species (ROS) and other oxidative substances, which could cause damage to cellular tissues [29]. Therefore, in response to oxidative stress, FG had developed more efficient antioxidant mechanisms than the SG group. In addition, the metabolomic analysis revealed a significant up-regulation of decanoyl-L-carnitine and taurine content in the FG group compared to the SG group. Research suggested that L-carnitine could improve T-AOC and SOD activity in the body [30]. Taurine is one of the important functional amino acids. Its main biological function is to regulate normal physiological functions of the body, enhancing antioxidant capacity. The previous study revealed a significant reduction in serum SOD and T-AOC levels in spotted seabass following a low-phosphorus diet. However, in the present study, the antioxidant capacity of FG increased when it was adapted to low-phosphorus conditions [22]. The findings suggested that the spotted seabass could increase its antioxidant capacity by boosting liver levels of decanoyl-L-carnitine and taurine, thereby enhancing the body’s ability to adapt to a low-phosphorus diet.

### 4.2. Fast-Growing Spotted Seabass Were Adapted to a Low-Phosphorus Diet Revealed in Transcriptomic Function

Transcriptomics sequencing revealed that compared to the FG, the liver of the FG exhibited up-regulation of DEGs mainly enriched in signaling pathways such as cytokine and cytokine receptor interactions, protein digestion and absorption, glycolysis and gluconeogenesis. This indicated the adaptive changes in immune response, energy metabolism, growth regulation, and nutrient absorption that occurred in the liver of spotted seabass fed a low-phosphorus diet. Among them, cytokines and cytokine receptors interacted and participated in processes such as host defense and cell death in congenital and adaptive inflammation [31]. The up-regulated DEGs in this pathway encompassed *INHBB*, *CCR3*, *CCR9*, and *CCL4*, all of which were important components of the immune system. They exerted regulatory control over the proliferation, differentiation, and migration of immune cells by binding to their corresponding receptors. This probably enhanced the immune system’s sensitivity and improved its immune capacity in spotted seabass. Additionally, this study revealed that compared to FG fed a low-phosphorus diet, SG exhibited up-regulation of gene expression levels for pro-inflammatory cytokines (*IL-1β*, *IL-8*, and *TNF-α*) in the liver. This indicated a more severe inflammatory response in the liver of SG, which was also the reason for their limited growth.

Steroid hormones regulate various physiological processes in vertebrates, such as sex differentiation, development, growth, and reproduction [32,33,34]. In this study, compared to SG, the expression of the *CYP2R1* gene was significantly up-regulated, while the expression of the *CYP24A1* gene was significantly down-regulated in the liver of FG. The former is a crucial enzyme involved the activation of vitamin D [35], while the latter is currently the only known enzyme that breaks down vitamin D [36,37]. Vitamin D is usually found in an inactive form in the body and needs to be metabolically activated to promote the absorption of calcium and phosphorus [38]. These results indicated that FG fed a low-phosphorus diet optimized the function of vitamin D in promoting calcium and phosphorus absorption by accelerating its activation and reducing its decomposition, thus supporting the rapid growth needs of spotted seabass. In addition, the comparative analysis of liver metabolites in different growth rates showed that the steroid hormone dehydroepiandrosterone (DHEA) was significantly up-regulated in the liver of FG. A previous study found that compared with the normal-phosphorus group, the differentiation and proliferation of osteoblasts were reduced when spotted seabass were fed a low-phosphorus diet [21]. Studies have shown that DHEA may promote bone health by promoting the growth of osteoblasts [39]. The above results suggested that the FG fed a low-phosphorus diet could promote the function of vitamin D and DHEA in the body of FG, thereby enhancing the reabsorption of calcium and phosphorus and improving bone health, which in turn promoted growth.

Glycolysis and gluconeogenesis play pivotal roles in the regulation of energy metabolism. In this study, compared to SG, the up-regulated DEGs of FG were enriched in glycolysis and gluconeogenesis pathways, including *GCK*, *ALDH3B1*, and *GAPDH*. Among them, *GCK* is a key enzyme in the glycolytic pathway. The upregulation of GCK in the liver of the FG could increase glucose phosphorylation efficiency, thereby expediting the glycolytic process and providing supplementary energy sources for its rapid growth. The optimization of glucose utilization in spotted seabass under phosphorus-limited conditions facilitated the fulfillment of their energy requirements for growth. Additionally, the up-regulation of *ALDH3B1* could facilitate the adaptation of FG to cope with the accumulation of potential toxic substances and metabolites in a low-phosphorus diet [40]. *GAPDH*, a pivotal enzyme in the glycolytic pathway [41,42], plays a crucial role in facilitating the glycolytic process and enhancing ATP production. Elevated levels of *GAPDH* indicate an augmented carbohydrate metabolism, thereby facilitating increased energy production and fostering muscle hypertrophy [43]. The expression of *GAPDH* in grass carp and several other fish suggests its potential as a reliable marker gene for carbohydrate metabolism [44]. Studies have shown that the glycolytic capacity of fish was up-regulated after feeding on a low-phosphorus diet [21]. This probably be attributed to the fact that fish necessitated higher energy expenditure for sustaining fundamental physiological processes in low-phosphorus conditions. The increase in the hepatic glycolysis and gluconeogenesis pathways in FG showed a pivotal metabolic strategy enabling their adaptation to low-phosphorus environments while sustaining rapid growth. These results provide valuable information about the key genes to use as biomarkers of growth in selective breeding programs for spotted seabass.

### 4.3. Fast-Growing Spotted Seabass Fed a Low-Phosphorus Diet Exhibited Greater Protein Digestion and Transfer Ability

The integrated analyses of metabolomics and transcriptomics revealed a marked enrichment of transcription-associated metabolites in the protein digestion and absorption pathways. The digestion and absorption of protein are directly correlated with the growth rate of fish. Compared to SG, amino acids (L-glutamine, L-methionine, L-tryptophan, L-phenylalanine, L-proline, L-threonine) were significantly up-regulated in the liver of FG. Research on grass carp (*Ctenopharyngodon idella*) has shown that L-tryptophan can increase GOT activity in the liver, enhancing the utilization efficiency of amino acids [45]. Moreover, L-methionine serves as a precursor for cysteine and is an essential component of proteins in animal bodies, improving the utilization of sulfur-containing amino acids [46]. L-threonine has emerged as a limiting amino acid that significantly impacts the growth performance of fish, as it can directly participate in protein synthesis as a substrate [47]. Moreover, L-glutamate promotes the synthesis of non-essential amino acids for body protein deposition [48,49]. Previous studies have found that compared to spotted seabass fed a normal-phosphorus diet, spotted seabass fed a low-phosphorus diet enhanced amino acid catabolism and lowered protein efficiency [21]. However, the increased contents of multiple amino acids in the FG group indicated a greater capacity for amino acid uptake and utilization, allowing it to utilize dietary protein more efficiently for body protein synthesis. The decline in amino acid content in the SG may be associated with increased application of amino acids in non-growth aspects, such as creatine synthesis. Research has shown that the biosynthesis of creatine requires three amino acids: arginine, glycine, and methionine [50]. Therefore, when the spotted seabass was not adapted to the low-phosphorus diet, it may have provided energy for basic life activities by synthesizing creatine from amino acids. In addition, in this study, the up-regulated DEGs (*SLC7A7; PRSS1*; *MEP1B*; *COL12A1*) in the FG group were also enriched in protein digestion and absorption compared with SG. The crucial role of *SLC7A7* lies in its function as an amino acid transporter, which is responsible for maintaining cellular balance and homeostasis through the transportation of amino acids across cell membranes [51,52,53]. *PRSS1* and *MEP1B* are two kinds of trypsin, which has been reported to lyse a variety of peptide and protein substrates [54,55]. The *COL12A1* gene is a collagenase involved in the structure of the extracellular matrix [56]. In this study, under phosphorus-deficient conditions, FG exhibited enhanced efficiency in amino acid transport, protein digestion ability, and liver tissue repair. In conjunction with the aforementioned findings, augmenting the amino acid content within the low-phosphorus diet could prove advantageous in acclimating to the low-phosphorus condition.

## 5. Conclusions

A combination of transcriptomics and metabolomics analyses was employed to investigate the changes in metabolites and genes between the SG and FG. Distinct pathways were identified in the liver of fast-growing spotted seabass, mainly up-regulating cytokine receptor interactions, glycolysis and gluconeogenesis, steroid biosynthesis, and protein digestion and absorption. Additionally, the present study revealed that the hepatic antioxidant levels of FG fed a low-phosphorus diet were notably elevated compared to SG. Moreover, a large number of metabolites in the liver of FG were significantly up-regulated, especially amino acids, L-carnitine, and dehydroepiandrosterone, suggesting that amino acid metabolism, steroid biosynthesis, and their related metabolites are potential biomarkers. In conclusion, the findings suggest that the fast growth advantage observed in spotted seabass fed a low-phosphorus diet can be attributed to their enhanced hepatic protein absorption, increased glucose metabolism, and heightened antioxidant capacity. These results provide valuable information about the key genes to use as biomarkers of growth in selective breeding programs for spotted seabass and contribute to our understanding of the molecular mechanisms and regulatory pathways of fish adaptation to growth under low-phosphorus conditions.

## Figures and Tables

**Table 1:** Formulation and proximate composition of the experimental diets (dry weight, %).

Ingredients	Content/%
Deboned fishmeal	15.00
Squid paste	3.00
Casein	22.00
Wheat gluten	8.00
Dextrin	37.00
Microcrystalline cellulose	2.52
Fish oil	9.00
Vitamin C	0.10
Vitamin premix ^a^	0.40
Mineral premix ^b^	0.50
Choline chloride	0.50
KCI	1.00
NaCl	0.78
Taurine	0.10
Y_2_O_3_	0.10
Total	100.0
Proximate composition	
Crude protein	45.76
Crude lipid	11.34
Total Ca	0.35
Total P	0.11

^a,b^ Mineral premix and vitamin premix were prepared according to Zhang et al. [5].

**Table 2:** Nucleotide sequences of the primers used to assay gene expressions by RT-qPCR.

Genes	Forward Primer (5′–3′)	Reverse Primer (5′–3′)
*SLC2A3*	CAGAAGAGGCCGAACACCAA	TGGCAGATTCGTACGGAAGAC
*APOE*	TGGTTTCACATCCCTCCTGC	CACCAAGATCCGTGAGCAGT
*IL-17*	GGTGTCCAACTACACGCTGA	TGCACCGGCTGGTAACTTAG
*AQP9*	AGTGATTGCCAGGATGCACA	CTGTCACTGGTGCAAATGCC
*CYP2R1*	CTCGGTGGCATCTTGACTGT	GCCAGTTTGCGGTGTTCAAT
*GCK*	CTGGCTTGTGGGGACAGATT	GAGGCTGGCCCTCTTTATCC
*IL-10*	ATGGGCGAACTGGATCTGC	TTAGGGTCAGCCGGTCTTCA
*COX-2*	ACTTTCACGACCACGCTCTAA	GCAGGGAGAACAGTTCAGACA
*TLR2*	TTGCCAAATGCAATCCCGAC	TCATCTTCAACCAGCGGTGT
*TNF-α*	GATCGTCATCCCACAAACCG	GCTTTGCTGCCTATGGAGTC
*IL-1β*	TCTGTGGCGCTGCTCTTAAA	TGCCCAGTGGAATGGACTTG
*IL-8*	TGGAGCTGATTCCTGCCAAC	TCCCGATCTGTTCAGGGTGT
*β-actin*	CAACTGGGATGACATGGAGA	AGTTGGCTTTGGGGTTCAGG

Abbreviations: *SLC2A3*: glucose transporter 3; *APOE*: apolipoprotein Eb; *IL-17*: interleukin-17; *AQP9*: aquaporin 9; *CYP2R1*: vitamin D 25-hydroxylase; *GCK*: glucokinase; *IL-10*: interleukin-10; *COX-2*: cyclooxygenase-2; *TLR2*: toll-like receptor 2; *TNF-α*: tumor necrosis factor α; *IL-1β*: interleukin-1β; *IL-8*: interleukin-8.

**Table 3:** Offline data statistics.

Sample	Reads No.	Bases (bp)	Q30 (bp)	N (%)	Q20 (%)	Q30 (%)
FG1	47,738,948	7,208,581,148	6,891,660,095	0.004106	98.43	95.60
FG2	61,344,676	9,263,046,076	8,860,529,909	0.004164	98.46	95.65
FG3	51,054,882	7,709,287,182	7,355,152,727	0.004161	98.35	95.41
SG1	50,271,034	7,590,926,134	7,247,952,498	0.004192	98.38	95.48
SG2	51,436,446	7,766,903,346	7,418,109,058	0.004164	98.38	95.51
SG3	47,522,776	7,175,939,176	6,851,975,975	0.004203	98.38	95.49

Note: Reads No.: total number of reads; Bases (bp): total number of bases; Q30 (bp): total number of bases with a base recognition accuracy rate of over 99.9%; N (%): percentage of ambiguous bases; Q20 (%): percentage of bases with a base recognition accuracy rate of over 99%; Q30 (%): percentage of bases with a base recognition accuracy rate of over 99.9%. FG: fast-growing spotted seabass; SG: slow-growing spotted seabass.

**Table 4:** Data filtering statistics.

Sample	Clean Reads No.	Clean Data (bp)	Clean Reads %	Clean Data %
FG1	46,736,204	7,049,662,557	97.90	97.80
FG2	60,107,352	9,065,731,624	97.98	97.87
FG3	49,882,034	7,521,750,060	97.70	97.57
SG1	49,122,294	7,406,405,066	97.71	97.57
SG2	50,279,510	7,582,177,575	97.75	97.62
SG3	46,469,094	7,001,358,704	97.78	97.57

Note: Sample: sample name; Clean Reads No.: number of high-quality sequence reads; Clean Data (bp): number of high-quality sequence bases; Clean Reads %: percentage of high-quality sequence reads among sequencing reads; Clean Data %: percentage of high-quality sequence bases among sequencing bases. FG: fast-growing spotted seabass; SG: slow-growing spotted seabass.

**Table 5:** Major differently expressed genes and related pathways.

Level 2	Pathway	KO
Carbohydrate metabolism	Glycolysis/gluconeogenesis	*GCK* (+); *AL3B1* (+); *GAPDH* (+)
Lipid metabolism	Steroid biosynthesis	*SOAT1* (+); *CYP2R1* (+); *CYP24A1* (−)
Digestive system	Protein digestion and absorption	*PRSS1*(+); *MEP1B* (+); *COL12A1* (+); *SLC7A7* (+); *SLC7A6* (−)
Endocrine system	Insulin secretion	*RYR2* (+); *GCK* (+); *SLC2A1* (+); *GLUC2* (−); *GLUC1* (−); *KCNJ11* (−)
Signaling molecules and interaction	Cytokine–cytokine receptor interaction	*INHBB* (+); *CCR3* (+); *CCR9* (+); *CCL4* (+); *GDF11* (−); *IL-10* (−)

Note: KO: the name of the gene in the KEGG database; “−”: compared with the SG group, the expression level of the mRNA in the FG group significantly down-regulated; “+”: compared with the SG group, the expression level of the mRNA in the FG group significantly up-regulated.

**Table 6:** Major different metabolites and related metabolic pathways.

Metabolite	VIP	FC	*p* Value	Metabolic Pathway	Trend
Decanoyl-L-carnitine	1.5732	2.0500	2.4030 × 10^−6^	—	↑
Dehydroepiandrosterone	1.5457	4.8000	8.8503 × 10^−6^	Steroid degradation	↑
Creatine	1.5676	0.6300	3.3865 × 10^−5^	Arginine and proline metabolism	↓
D-Galactose	1.4958	1.3800	1.8468 × 10^−3^	Mineral absorption	↑
L-Methionine	1.4611	1.4000	4.8716 × 10^−3^	Central carbon metabolism	↑
L-Glutamine	1.4409	1.2000	7.2854 × 10^−3^	Protein digestion and absorption	↑
L-Threonine	1.4388	1.1700	1.0996 × 10^−2^	Mineral absorption Protein digestion and absorption	↑
L-Tyrosine	1.5135	1.4800	1.1841 × 10^−2^	Protein digestion and absorption	↑
L-Phenylalanine	1.4556	1.3700	3.0524 × 10^−2^	Mineral absorption Protein digestion and absorption	↑
L-Proline	1.4665	1.6500	3.4051 × 10^−2^	Mineral absorption Protein digestion and absorption	↑
Taurine	1.3846	1.19	0.017041367	Neuroactive ligand–receptor interaction	↑

Note: “↑”: compared with the SG group, metabolites in the FG group significantly increased; “↓”: compared with the SG group, metabolites in the FG group significantly decreased.

## Data Availability

The data presented in this study are available upon request from the corresponding author.
